# Dynamic Parameter Identification for a Manipulator with Joint Torque Sensors Based on an Improved Experimental Design

**DOI:** 10.3390/s19102248

**Published:** 2019-05-15

**Authors:** Jidong Jia, Minglu Zhang, Xizhe Zang, He Zhang, Jie Zhao

**Affiliations:** 1 School of Mechanical Engineering, Hebei University of Technology, Tianjin 300130, China; jiajidong.0724@163.com; 2 State Key Laboratory of Robotics and System, Harbin Institute of Technology (HIT), Harbin 150006, China

**Keywords:** dynamic parameter identification, excitation optimization, maximum likelihood estimation, robotics, motion control, experiment design, signal processing

## Abstract

As the foundation of model control, robot dynamics is crucial. However, a robot is a complex multi-input–multi-output system. System noise seriously affects parameter identification results, thereby inevitably requiring us to conduct signal processing to extract useful signals from chaotic noise. In this research, the dynamic parameters were identified on the basis of the proposed multi-criteria embedded optimization design method, to obtain the optimal excitation signal and then use maximum likelihood estimation for parameter identification. Considering the movement coupling characteristics of the multi-axis, experiments were based on a two degrees-of-freedom manipulator with joint torque sensors. Simulation and experimental results showed that the proposed method can reasonably resolve the problem of mutual opposition within a single criterion and improve the identification robustness in comparison with other optimization criteria. The mean relative standard deviation was 0.04 and 0.3 lower in the identified parameters than in *F*_1_ and *F*_3_, respectively, thus signifying that noise is effectively alleviated. In addition, validation experimental curves were close to the estimation model, and the average of root mean square (RMS) is 0.038, thereby confirming the accuracy of the proposed method.

## 1. Introduction 

A manipulator is a complicated multi-input–multi-output (MIMO) system, which has strongly coupled nonlinear characteristics. Given structured and unstructured uncertainties, such as elastic deformation, assembly clearance, inertia, Coriolis, gravity, and friction torque, the repetitive positioning accuracy of a robot and the perceived accuracy of joint torque information are seriously affected. Most robot manufacturers do not provide related information or partial parameters to obtain an accurate dynamic model [1,2]. Therefore, experimental identification is the optimal choice for obtaining these types of information. However, sensor measurement and process noise will challenge experimental identification. To mitigate the effects of nonlinearity and improve the advanced model-based control performance, a complete noise reduction system must be considered to perform the accurate estimation of dynamic parameters, including statistics of noise characteristics, excitation signal optimization, and noise processing.

Some researchers have provided significant contributions to the model of robot dynamics identification. Gautier constructed a model based on energy identification in [3] and a power model in [4], depending only on the functions of joint position and velocity to avoid introducing acceleration significantly, thereby leading to including non-negligible noise in the estimation process. A direct inverse dynamic identification model, which is based on an output error, has been proposed in [5]; this model solely requires force/torque measurement to substitute a usual position output. The most common model of identification is inverse dynamic identification model (IDIM), which provides more information than other models. In addition, the model can be linearly formulated with respect to a set of minimal dynamic parameters, thereby allowing us to construct a well-conditioned over-determined regression matrix [6,7,8].

Subsequently, parameters are estimated through numerical optimization based on the abovementioned models. The least squares (LS) method [9] and maximum likelihood estimation (MLE) [10] technique are the typical robot parameter identification methods under the class of estimation algorithms. Moreover, these techniques can be expanded in several ways, such as the weighted least squares (WLS) estimation method [11] and nonlinear least squares (NLS) optimization method [5]. Another common alternative estimation approach is based on the Kalman filter and its expansions, such as the extended Kalman filter (EKF) and unscented Kalman filter [12]. Gautier compared the WLS method with EKF estimation for parameter identification of a two degrees-of-freedom (DOF) robot in [13]; the estimation results obtained through the two techniques were similar, but the EKF was sensitive to initial conditions, and its convergence velocity was slower than that of the WLS method. With the further improvement of instrument variable theory, some researchers have aimed to bridge the gap between theory and control engineering practices and complete the MLE method with profound statistical characteristics in [14,15,16]. These attempts have undeniably achieved favorable identification effects, but the choice of auxiliary variables is significantly affected by system control law. Moreover, several other identification methods for a manipulator are based on the genetic algorithm (GA) in [17] and neural networks to improve the accuracy of estimation in [18,19]. Detailed summaries of these methods can be found in [6,20] for further understanding.

Furthermore, an experimental design is a crucial link in a closed-loop identification process, because the noise on joint position and output torque measurements, friction, and other unmodeled effects seriously affect the accuracy of estimated parameters. To reduce the bias and obtain favorable results, the exact statistical properties of the uncertainty terms must be determined. An appropriate experiment is designed by reducing the sensitivity of the system to noise and minimizing the values of the variances of the estimated parameters [14,16]. This process consists of two parts, namely, excitation trajectory design and optimization criterion. In [10,21], the closed-loop identification process is combined with an optimal excitation trajectory using parameterized finite Fourier series. The optimization process implements an iterative procedure until the Cramér–Rao lower bound is reached. Atkeson [22] proposed an excitation trajectory of a fifth-order polynomial in a joint space. Moreover, the excitation signal based on modified Fourier series [2], the finite sum of harmonic sine functions [17], and the function formed by combining several ways [23] are extended well. Two main optimization criteria are adopted; one criterion is based on minimizing the condition of an observation matrix [24]; the other criterion is to minimize the variations in the Fisher information matrix [10,21]. However, noise exists in the input and output, but most optimization technologies are justified by observing a one-sided error and cannot perform full optimization. In addition, considering the time cost in the optimization process, Jingfu [25] proposed a new trajectory design method based on Hadamard’s inequality. This method is certainly a good innovation, but time is relatively loose and seems insignificant in offline optimization identification.

The present research aims to realize the dynamic parameter identification of a manipulator as accurately as possible. The primary contribution is that an improved optimization algorithm is proposed on the basis of the multi-criteria embedded nonlinear optimization method to design the exciting trajectory for minimizing the impact of noise. Considering the influence of a certain criterion on solving the parameter identification, our approach aims to reduce the condition of the observation matrix and determinant of the Fisher information matrix simultaneously in a nonlinear optimization process. In the present research, the model that will be considered is based on IDIM and is linear in the parameters to be identified, thereby implying that the input and output of the robot correspond to the output and input functions of the identification model. In addition, extracting the base set of the parameters is advantageous. Furthermore, the appropriate weight values are obtained on the basis of the statistical characteristics of noise. All joints of the robot are designed simultaneously in the identification procedure to solve the MIMO coupled problem. The simulation and experimental results show that this method can identify parameters accurately and is superior to the other nonlinear optimization methods in terms of robustness, time consumption, and other factors. In addition, the proposed optimization method is not limited to a series robot but also applicable to the optimized experimental design of parallel and exoskeleton robots. This condition is due to the fact that, to obtain a high-precision dynamic model, the input must fully stimulate the motion form of the system to ensure that the experimental data can fully reflect the physical characteristics of the system, and a good excitation design can reduce the impact of noise on the results. Examples of this include system identification of aircraft, parameter identification of sorting robots, friction identification, force-free control and collision detection.

The remainder of this paper is organized as follows. Section 2 introduces the development of a dynamic linear model, and the MLE method is used to estimate the robot parameters. Section 3 presents the different optimization criteria and describes the process of obtaining optimal excitation trajectories based on a single criterion and with the proposed multi-criteria embedded nonlinear optimization method. Section 4 discusses the identification experiment process and compares the traditional method with the proposed algorithm. Section 5 analyzes the relevant experimental results. Section 6 provides some conclusions drawn from this research.

## 2. Modeling and Identification of Robot Parameters

**Figure 1:**
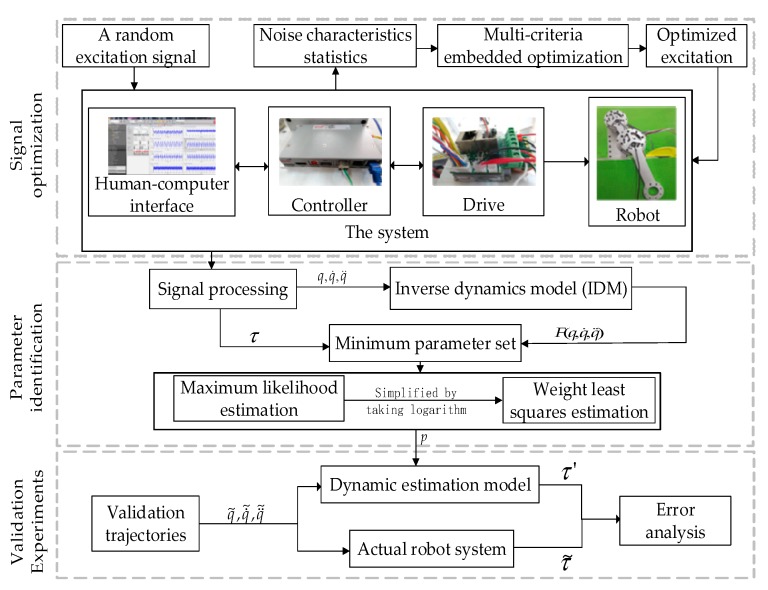
Overall procedure of parameter identification.

In this section, we obtained the identification procedure using the IDIM and the WLS estimation technologies to identify the base parameters of a multi-jointed robot. To reduce the system noise, we used mean and low pass filtering based on a periodic excitation trajectory to improve the signal-to-noise ratio. Moreover, we could obtain the estimated results using the linearized formulation model. The specific identification procedure is illustrated in Figure 1.

### 2.1. Dynamic Identification Model

Generally, we can determine the dynamic model of an n-joint rigid robot based on the Euler–Lagrange or the Newton–Euler formulation in the joint space as

(1) $$M(q)\ddot{q}+C(q,\dot{q})\dot{q}+G(q)+\tau_{f}=\tau$$

where $M(q)\in {ℜ}^{n\times n}$ is the inertia matrix, $C(q,\dot{q})\in {ℜ}^{n\times n}$ contains Coriolis and centripetal matrix, $G(q)\in {ℜ}^{n}$ is the vector of the gravitational matrix, $\tau_{f}\in {ℜ}^{n}$ is the friction force term, $\tau \in {ℜ}^{n}$ is the input torque vector to the system, and $q\in {ℜ}^{n}$ is the vector of relative generalized coordinates used to model the system, $\dot{q},\ddot{q}\in {ℜ}^{n}$ represents the generalized velocities and accelerations based on differentiation of *q*. The friction forces are described as [26]

(2) $$\tau_{f}=F_{c}\mathrm{sgn}(\dot{q})+F_{v}\dot{q}$$

where $F_{c},F_{v}\in {ℜ}^{n\times n}$ are the diagonal matrices that describe the coefficient of Coulomb and viscous friction, correspondingly.

The mathematical model (1) in a linear parameterized form that depends on a set of parameters must be rewritten for dynamic parameter identification. Modified Denavit and Hartenberg [27] convention can help us obtain the linear model with *N_s_* standard parameters.

(3) $$\tau =\phi_{s}(q,\dot{q},\ddot{q})\theta_{s}$$

where $\phi_{s}(q,\dot{q},\ddot{q})\in {ℜ}^{n\times N_{s}}$ is the regression matrix of a nonlinear function of joint position, velocity, and acceleration vectors, and $\theta_{s}\in {ℜ}^{N_{s}\times 1}$ is the vector of standard parameters to be estimated. A total of 12 standard parameters are used by each link and joint for rigid robots, which contain the mass *m_j_* of each link *j*, the first mass $\left(\begin{array}{ccc} mx_{j} & my_{j} & mz_{j} \end{array}\right)$ moments of link *j*, the six components of the inertia tensor of link j at the origin of frame *j*, $\left(\begin{array}{cccccc} Ixx_{j} & Ixy_{j} & Ixz_{j} & Iyy_{j} & Iyz_{j} & Izz_{j} \end{array}\right)$ and the coefficients of viscous and Coulomb friction torque of joint *j* $(\begin{array}{cc} F_{c_{j}} & F_{v_{j}}) \end{array}$.

The factor that truly affects robot dynamics is only part of the inertial parameters and can be estimated through identification procedures. The set of a minimum number of inertial parameters, that is, the base parameter set, can be selected on the basis of a numerical method with respect to the QR decomposition [28] or by regrouping the standard parameters through linear relations [29,30]. The dynamic equation (3) can then be regrouped into another form with *N_b_* identifiable base parameters.

(4) $$\tau =\phi_{b}(q,\dot{q},\ddot{q})p_{b}$$

where $\phi_{b}(q,q,q)\in {ℜ}^{n\times N_{b}}$ is a subset of the regression matrix $\phi_{s}$, and $p_{b}\in {ℜ}^{N_{b}}$ is the base parameters by regrouping.

### 2.2. Data Acquisition and Signal Processing

In fact, the known system must be given continuous excitation signals to demonstrate the mechanical and physical properties fully. Although the observation matrix *ϕ_b_* has full rank, and *p_b_* can be obtained through the pseudo-inverse matrix from Equation (4), the solutions of identification are local and unreliable given the complex modeling errors, measurement noise, friction, and other factors. The ideal dynamic model is inexistent. Thus, assuming that the fundamental frequency *ω_f_*, sampling frequency *ω_s_*, and the *k*th sampling time as *t_k_* of the excitation trajectory are known, Equation (4) can be extended in an over-determined set with *M* = *ω_s_*/*ω_f_* measurement points over one period *T* as follows:

(5) $$\Gamma =F(q,\dot{q},\ddot{q})p_{b}+\varepsilon$$

where $\Gamma \in {ℜ}^{nM\times 1}$ is the measured force/torque vector; $F(q,\dot{q},\ddot{q})\in {ℜ}^{nM\times N_{b}}$ is the observation matrix, also called the global information matrix; $\varepsilon \in {ℜ}^{nM}$ is the zero mean random vector of errors derived from the term of nonlinear noise.

Considering that the collected data (e.g., position and torque data) contain noise, it is a fundamental step to reducing the influence of noise on τ and F for improving the accuracy of parameter identification results. Here, velocity and acceleration are the numerical differentiation with respect to the position *q*. In the process of data collection, given the periodicity of excitation trajectory, we combined the mean filtering algorithm and a second-order low-pass digital Butterworth filter to process signal noise, especially acceleration and torque.

### 2.3. MLE for Parameter Estimation

**Figure 2:**
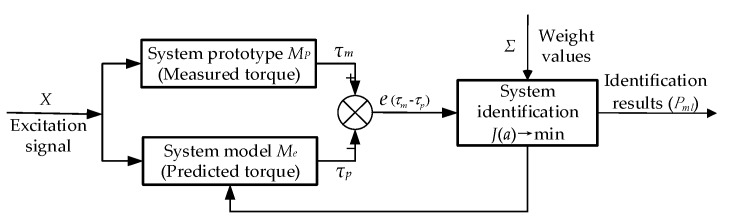
*P_ml_* identification schematic of weighted least squares (WLS).

System identification aims to determine a system model based on input and output data from a specified class of models, thereby making it equivalent to the estimated system. The principle is depicted in Figure 2. The same signal *X* is used to excite the system prototype *M_p_* and the system model *M_e_*. Moreover, the output signals are *τ_m_* and *τ_p_*, respectively, and the error is *e*. An error criterion function *J*(*a*) = *e^T^*Σ*e*, which can be taken as the function of the error, is specified to modify the parameter vector *a*. Recursion is repeated until the error criterion *J*(*a*) is minimal.

Robot identification handles the problem of estimating the model parameters from the measured ones based on a statistical framework during a robot excitation experiment. Assuming that the measured joint position *q_m_*(*k*) and torque *τ_m_*(*k*) at *k*th sampling time conform to a random zero-mean Gaussian noise *ε_q_*(*k*), *ε_τ_*(*k*), i.e.,

(6) $$\begin{array}{c} q_{m}(k)=q(k)+\varepsilon_{q}(k)\\ \tau_{m}(k)=\tau (k)+\varepsilon_{\tau }(k) \end{array}$$

where *ε_q_*(*k*) and *ε_τ_*(*k*), i.e., *ε_q_*(*k*) + *ε_τ_*(*k*) = *ε*, are integrated to satisfy Equation (5). The noise-free version of these variables satisfies Equation (4).

From the perspective of statistics, the MLE method is conducted for the dynamic parameters of the robot [10]. First, a likelihood function of joint angles *q_m_*(*k*) and joint moment *τ_m_*(*k*) measurement value is constructed. Second, the MLE of parameter vector *p* is obtained by maximizing the function. Given that the noise on different measurements are independent of each other and obey the Gaussian distribution, the following quadratic cost function can be minimized:

(7) $$L(q_{m},\tau_{m}|p)=\frac{1}{2}\sum_{k=1}^{M}\sum_{j=1}^{n}\left(\frac{\varepsilon_{q_{j}}^{2}(k)}{\sigma_{q_{j}}^{2}}+\frac{\varepsilon_{\tau_{j}}^{2}(k)}{\sigma_{\tau_{j}}^{2}}\right)$$

where $L(q_{m},\tau_{m}|p)$ is the likelihood function, *ε_qj_*(*k*) and *ε_τj_*(*k*) are the noise on the joint position and torque of the *j*th joint, respectively. $\sigma_{q_{i}}^{2}$ and $\sigma_{\tau_{i}}^{2}$ are their corresponding variances. Detailed steps for determining these variables can be obtained in [31].

Considering Equation (4), the minimization problem of Criterion (7) will be transformed into an NLS optimization problem, thereby implying the necessity to obtain the *ε_qj_*(*k*) and *ε_τj_*(*k*) of every estimated parameter *p* with respect to the given measured data *q_m_*(*k*) and *τ_m_*(*k*) in practical implementation. This process is nearly infeasible using the present formulation. However, we can find that, in the actual experimental process, the noise level of the measurement value is much lower in the joint angle than in the actuator force of the joint. Therefore, no noise and error may be realized in measuring the joint angle. At this time, the maximum likelihood estimator of the parameters can be obtained by maximizing the likelihood function. Considering that the maximum likelihood function is equivalent to the logarithm of the likelihood function, the maximum likelihood parameter estimation method can be significantly simplified by taking the logarithm of the original likelihood function. Under this assumption, the MLE method reduces to the linear WLS estimation algorithm, whose weighted function is the inverse of the noise to the standard deviation of the torque value of the measured actuator [31].

(8) $$p_{ml}={\left(F^{t}\Sigma^{-1}F\right)}^{-1}F^{t}\Sigma^{-1}\Gamma$$

where

(9) $$F=\left[\begin{array}{c} \phi {\left(q_{m}(1),{\dot{q}}_{m}(1),{\ddot{q}}_{m}(1)\right)}_{n\times N_{b}}\\ \phi {\left(q_{m}(2),{\dot{q}}_{m}(2),{\ddot{q}}_{m}(2)\right)}_{n\times N_{b}}\\ \vdots \\ \phi {\left(q_{m}(M),{\dot{q}}_{m}(M),{\ddot{q}}_{m}(M)\right)}_{n\times N_{b}} \end{array}\right]$$

(10) $$\Gamma =\left[\begin{array}{c} \tau_{m}{\left(1\right)}_{n}\\ \tau_{m}{\left(2\right)}_{n}\\ \vdots \\ \tau_{m}{\left(M\right)}_{n} \end{array}\right]$$

and Σ is the diagonal covariance matrix of the measured actuator torques such that $\Sigma =diag\left(\begin{array}{ccccc} \sigma_{1}^{2}I_{M} & \ldots & \sigma_{j}^{2}I_{M} & \ldots & \sigma_{n}^{2}I_{M} \end{array}\right)$, where *I_M_* is the (*M* × *M*) identity matrix [30]. The covariance matrix of MLE *p_ml_* is equal to

(11) $${\left(F^{T}\Sigma^{-1}F\right)}^{-1}$$

Furthermore, if all the noise of the measured actuator torque has the same standard deviation, then the MLE will further degrade to the standard linear LS method (e.g., $p_{ls}={\left(F^{t}F\right)}^{-1}F^{t}\Gamma$). This result certainly loses the significance of the statistical framework.

## 3. Obtaining the Optimal Robot Excitation Trajectories

The optimal experiment design refers to the problem with respect to a nonlinear optimization with motion constraints, that is, generally, constraints on joint angles, velocities, and accelerations, and on the robot end effector positions in the Cartesian space to avoid collision with the environment and with itself. In addition to the generality, some researchers have added other conditions to the constraints, such as singular values of the observation matrix [32]. Given the actuator displacement as the control input, a criterion must be designed to represent the sensitivity of the input and output variables with respect to the solution of the estimate parameters. Considering the interaction between different optimization criteria, our main contribution is to propose a multi-criteria embedded nonlinear optimization method to obtain the optimal excitation.

### 3.1. Optimal Criteria for the Experiment Design

Common optimization problems are based on the deterministic framework and obtain the solution of dynamic parameters by minimizing the error term (i.e., ε in Equation (5)). To equilibrate the disturbance influence of the input and output noise on the parameter estimates, the condition number of the regression matrix is typically selected as the optimization objective function [2]. Gautier and Khalil [24] optimized a linear combination with a factor for equilibrating the observation matrix using this criterion and a fifth-order polynomial trajectory. Armstrong [33] described the optimization design of the experiment trajectory based on maximizing the minimum singular value of the square matrix *F^T^F*; some ways to expand on this condition have been used in [17,32]. Jingfu [25] proposed an approach by using the trace of the square matrix *F^T^F* based on Hadamard’s inequality. The collection of these methods can be found in [24,34].

Another common optimization problem is based on a statistical framework, and its most prominent feature is that the statistical characteristics of noise are considered in the optimization process. The representative one is the optimal objective function of the deformation of the Fisher information matrix (e.g., (*F^T^*Σ^−1^*F*)^−1^) presented by Swevers [10] for the MLE parameter identification of a serial robot. Afterward, it has been applied for a parallel robot [35]. Subsequently, many improvements and applications based on this criterion can be found in [15,21,23,31,36].

Notably, a new criterion based on a multi-objective optimization method is proposed in [37]. The main contribution of this approach is to find trajectories with favorable properties in accordance with more than a single criterion. This method solves the uncertainty of some solutions when the lower boundary is non-convex, but it must obtain the appropriate optimization weight value of the objective function in advance, thus undoubtedly increasing the computation and easily falling into the local solution of multiple optimizations.

From the linearized parameter model (5), we can determine that the condition number of the regression matrix represents the influence of the disturbance on the identification solution, and the physical meaning represented by the determinant value of the covariance matrix is the volume of the area of the maximum probability density function of the parameter to be estimated. Given the abovementioned characteristics, we propose a multi-criteria embedded optimization method, in which the optimization criteria (−log(det(*F^T^*Σ^−1^*F*)^−1^)) are embedded in the constraint conditions, and the interaction between the two criteria is compromised. In addition, the embedded optimization method makes the dual objective function optimized simultaneously in the optimization iteration process, thus avoiding the sensitivity of the multi-objective optimization approach to the goal weight value. The physical meaning is to obtain a regression matrix with good “behavior” when the solution of the estimated parameters is relatively determined, so as to reduce the sensitivity of identification results to interference.

The abovementioned optimization criteria are summarized in Table 1. Next, we will obtain the excitation trajectory based on our proposed optimization method.

### 3.2. Optimal Excitation Signal for the Experiment Design

Swevers proposed the excitation trajectory based on a finite Fourier series in [10]. The trajectory for each joint is a finite sum of *N* harmonic sine and cosine functions. The main advantages of these functions are that: First, they satisfy the numerical average in the time domain due to periodicity and conveniently improve the signal-to-noise ratio of the measurement. Second, the velocity and acceleration can be calculated using the position vector in analytical forms without using the numerical differentiation method. In addition, the effect on robot flexibility can be avoided by selecting an appropriate frequency bandwidth. The specific form for the *j*th of an n-link robot is defined as follows

(12) $$\begin{array}{l} q_{j}(t)=\sum_{l=1}^{N}\frac{a_{j,l}}{\omega_{f}l}\sin(\omega_{f}lt)-\sum_{l=1}^{N}\frac{b_{j,l}}{\omega_{f}l}\cos(\omega_{f}lt)+q_{j0}\\ {\dot{q}}_{j}(t)=\sum_{l=1}^{N}a_{j,l}\cos(\omega_{f}lt)+\sum_{l=1}^{N}b_{j,l}\sin(\omega_{f}lt)\\ {\ddot{q}}_{j}(t)=\omega_{f}\sum_{l=1}^{N}b_{j,l}l\cos(\omega_{f}lt)-\sum_{l=1}^{N}a_{j,l}l\cos(\omega_{f}lt) \end{array}$$

where *j* = 1,2. *t* ϵ [0, *T*] with *T* is the period cycle time, *ω_f_* is the fundamental frequency, and *ω_f_* = 2*π*/*T*. *N* is the number of harmonics, and *q*_*j*0_ is the position offset of the joint of the excitation reference trajectory. In Reference [31], the fundamental frequency *ω_f_* is considered as a variable of the optimization problem to be added to the optimization design of the excitation trajectory, but the optimization results are not reflected in this process, so the selected variables are inappropriate. The base frequency must be in a good bandwidth range (e.g., the so-called bandwidth, which reflects the noise filtering characteristics of the system and also used to measure the transient response performance of the system). The trade-off of selecting the fundamental frequency is discussed in [10], it cannot be used as a variable in this optimization process. Here, 2*N* + 1 parameters are applied as the variables for the trajectory optimization problem of per joint. All variables include the Fourier coefficients of two joints (e.g., *q*_*i*0_, *a_l_* = [*a_j,l_*,…,*a_j,N_*], *b_l_* = [*b_j,l_*,…,*b_j,N_*]).

Various optimization criteria are listed in Table 1. Considering the interaction between single different optimization criteria and combining with the actual physical meaning representation, the condition number of the regression matrix is adopted as the optimization objective function. In order to make the identification results insensitive to noise, the optimization problem is actually expressed as searching suitable values in constraint domain to minimize the objective function:

objFunc = *F*_4_ = minimize Cond (*F*) (13)

Generally, the constraint domain mainly depends on the kinematics and geometric information of the robot system, including the position, velocity, acceleration information of the actuator and start and end points of joints. However, the optimization results are often affected by singular solutions in the optimization process, and it is easy to fall into the local optimum. In order to avoid these situations, we add *F*_2_ = (−log(det(*F^T^*Σ^−1^*F*)^−1^)) to the constraint as an additional constraint. Thereby, the excitation trajectory must be subjected to the following constraints:

(14) $$\left\{ \begin{array}{l} (-\log(\det{\left(F^{T}\Sigma^{-1}F\right)}^{-1}))<\omega_{2};\\ \begin{array}{cc} \left|q_{j}(t)\right|\leq q_{\max} & \forall i,t\\ \left|{\dot{q}}_{j}(t)\right|\leq {\dot{q}}_{\max} & \forall i,t\\ \left|{\ddot{q}}_{j}(t)\right|\leq {\ddot{q}}_{\max} & \forall i,t\\ q_{j}(t_{0})=q_{j}(t_{f})=0 & \forall i,t\\ {\dot{q}}_{j}(t_{0})={\dot{q}}_{j}(t_{f})=0 & \forall i,t\\ {\ddot{q}}_{j}(t_{0})={\ddot{q}}_{j}(t_{f})=0 & \forall i,t \end{array} \end{array}\right.$$

where *ω*_2_ represents the constraint target value of *F*_2_, which is derived from the objective value obtained when only *F*_2_ is used as the optimization objective function; $q_{\max}$ (rad), ${\dot{q}}_{\max}$ (rad s^−1^), and ${\ddot{q}}_{\max}$ (rad s^−2^) denote the bounds of joint position, velocity, and acceleration, respectively; and *t*_0_ and *t_f_* correspond to the initial and the end times; these constraints indicate that the position, velocity, and acceleration of the robot at the initial and end points of the reference trajectory are equal to 0, otherwise a strong tremor of the system will result and affect the accuracy of the parameters. Objective values are updated iteratively until the condition number of the regression matrix converges to the minimum and the optimization results satisfy the whole constraint space, the optimization terminates. At this point, the multi-objective embedded optimization method has been established.

## 4. Identification Implementation Process

**Figure 3:**
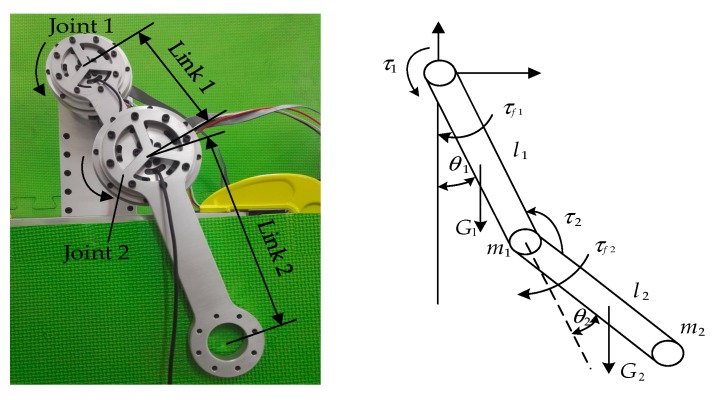
Prototype and simplified model of the two degrees-of-freedom (2-DOF) robot.

A planar two degrees-of-freedom (2-DOF) manipulator was adopted to evaluate the effectiveness of the method, which is designed here, as demonstrated in Figure 3. Compared with a single joint, the 2-DOF manipulator considers coupling factors, and its method is applicable to the parameter identification of additional DOFs. The considered manipulator has two revolute joints equipped with brushless motors produced by Maxon, which is equipped with an encoder for position measurements. The output end of the motor is equipped with a harmonic reducer at a reduction ratio of 80, and the output end of the reducer is equipped with a spoke-type torque sensor. To protect the torque sensor from axial force crosstalk, deep-groove ball bearings and thin-wall cross roller bearings are simultaneously equipped at the connection between the link and joint for unloading the undesired load. The control unit of the robot is constituted by Platinum Maestro Network Motion Controller based on EtherCAT, which is produced by Elmo, and the control algorithms are implemented in C++. The joint coordinates are defined in accordance with Denavit–Hartenberg notation and collected in vector *q* = [*q*_1_ *q*_2_]*^T^*, where *q_i_* represents the angular position of joint *j*. The command torque vector is defined as *τ* = [*τ*_1_ *τ*_2_]*^T^*, where *τ_i_* represents the torque applied to joint *j*.

### 4.1. Dynamic Model of the Planar Manipulator

The base parameter model of this robot contains five parameter combinations of the links with respect to the z_0_-axis and the first-order moments of the second link based on applying the dynamic formulation derived by Craig [38]. The friction that is modeled only in prismatic joints (independent generalized coordinates) given the friction in spherical and rotational joints (dependent generalized coordinates) can be neglected [39]. Therefore, we can obtain the vector *p_b_* of the identifiable parameters using regrouping standard parameters presented as follows:

$$p_{b}={\left[\begin{array}{ccccccccc} (m_{1}+m_{2})d_{1}^{2}+m_{2}d_{2}^{2} & m_{2}d_{1}d_{2} & m_{2}d_{2}^{2} & (m_{1}+m_{2})gd_{1} & m_{2}gd_{2} & F_{v1} & F_{c1} & F_{v2} & F_{c2} \end{array}\right]}^{T}$$

Following the Newton–Euler approach, the planar manipulator dynamic model is expressed in Equation (4). From this equation, we can determine the regression matrix, including

$$F=\left[\begin{array}{ccccccccc} {\ddot{q}}_{1} & c_{2}(2{\ddot{q}}_{1}+{\ddot{q}}_{2})-s_{2}(2{\dot{q}}_{1}{\dot{q}}_{2}-{\dot{q}}_{2}^{2}) & q_{2}^{2} & s_{1} & s_{12} & {\dot{q}}_{1} & \mathrm{sgn}({\dot{q}}_{1}) & 0 & 0\\ 0 & c_{2}{\ddot{q}}_{1}+s_{2}{\dot{q}}_{1}^{2} & {\ddot{q}}_{1}+{\ddot{q}}_{2} & 0 & s_{12} & 0 & 0 & {\dot{q}}_{2} & \mathrm{sgn}({\dot{q}}_{2}) \end{array}\right],$$

where *s_i_* = *sin*(*q_i_*) and *c_i_* = *cos*(*q_i_*).

### 4.2. Simulation of the Excitation Trajectory Optimization

Several kinds of excitation trajectories are obtained by the optimization design of condition number (Cond(*F*)) as a criterion or (−log(det(*F^T^*Σ^−1^*F*)^−1^)) as the criterion or based on multi-objective optimization as the criterion. For each optimization, the trajectory is parameterized through five-term Fourier series, yielding 11 parameters for each joint (e.g., *N* = 5). The fundamental frequency of the trajectories is 0.05 Hz, and the sampling rate of the simulation is 100 Hz. The length of the data sequence M is 2000 data in one period. The optimization process was performed in MATLAB R2017a environment using the nonlinear constrained optimization tools “Fmincon” and “Fgoalattain”. According to the motor parameters and motion constraints, the optimization constraints are set as follows:

- Embedded criterion *F*_2_ = (−log(det(*F^T^*Σ^−1^*F*)^−1^)) < −75;
- Joint angle limits (rad): −3.14 < *q*_1_ < 3.14, −3.14 < *q*_2_ < 3.14;
- Joint velocity limits (rad/s): −5.2 < ${\dot{q}}_{1}$ < 5.2, −5.2 < ${\dot{q}}_{2}$ < 5.2;
- Joint acceleration limits (rad/s^2^): −4.5 < ${\ddot{q}}_{1}$ < 4.5, −4.5 < ${\ddot{q}}_{2}$ < 4.5;
- The position, velocity, and acceleration of the two joints at the initial and end times are 0. e.g., $q_{j}(0)={\dot{q}}_{j}(0)={\ddot{q}}_{j}(0)=q_{j}(20)={\dot{q}}_{j}(20)={\ddot{q}}_{j}(20).$

**Figure 4:**
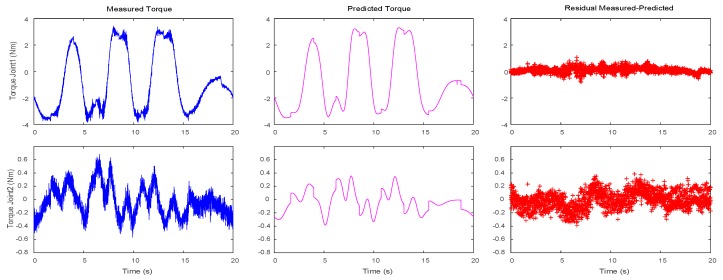
Random trajectory identification results for obtaining the noise characteristics.

In addition, the covariance matrix Σ in the optimization process was derived from a random trajectory, and the parameter was estimated through the traditional linear LS method (refer to [30] (p. 294)), as plotted in Figure 4. This process aims to collect the noise characteristics of the system, that is, σ_1_ = 5.0832 N^2^m^2^, and σ_2_ = 0.0499 N^2^m^2^. The target values of the multi-objective optimization criterion were obtained from the optimization results of the former criterion. In Table 2, we selected *ω*_1_ = 8 and *ω*_2_ = −75.

To avoid special situations, the results of each criterion for four selected trajectories are summarized in Table 2. When solving the optimization problem with the *F*_1_ criterion, the values of *F*_2_ were higher than those obtained when only *F*_2_ was used. Furthermore, the opposite occurs when the optimization problems were solved by *F*_2_. Moreover, the optimization process is easily trapped in the local minimum. This phenomenon had been resolved, that is, it will not significantly increase the value of other criteria when the relevant appropriate solutions are obtained. Either by using a weighted objective function to convert two objective functions in a single function as provided in [24,33] or by using a multi-objective optimization procedure based on the goal programming in [37] (the results of multi-objective optimization are shown in the last two columns of Table 2). However, the optimization results are sensitive to the goal values given the value setting of 2-DOF, as listed in Table 3. Therefore, a multi-criteria embedded (e.g., *F*_4_) nonlinear optimization method is considered.

### 4.3. Multi-criteria Embedded Nonlinear Optimization

According to Equation (14), the *F*_2_ criterion is added to the constraint condition. The middle two columns of Table 3 show the results of the multi-criteria embedded optimization. In this table, this method is mainly affected by the weight values in the constraint conditions and reduces the DOF in comparison with the multi-objective optimization criteria. In addition, the optimization method is equivalent to introducing penalty function into the objective function, thereby slightly improving the robustness, and this approach has a clear physical meaning.

**Figure 5:**
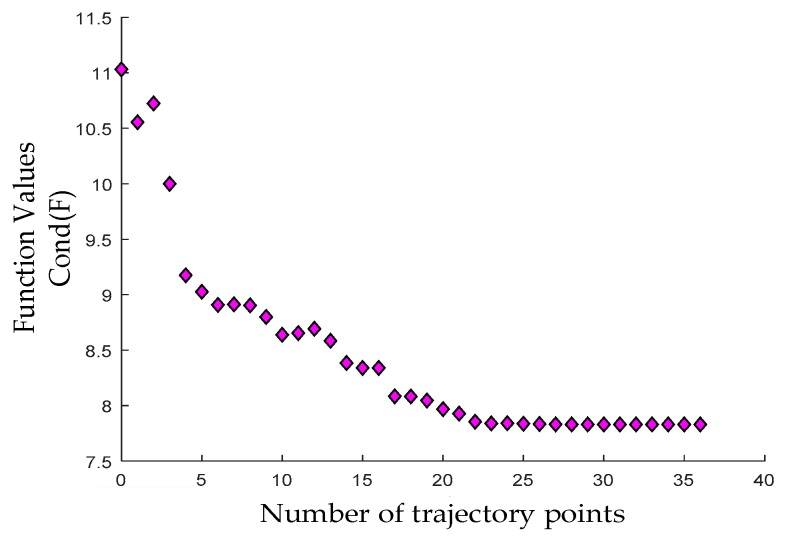
Condition number versus trajectory points for the actual 2-DOF manipulator.

**Figure 6:**
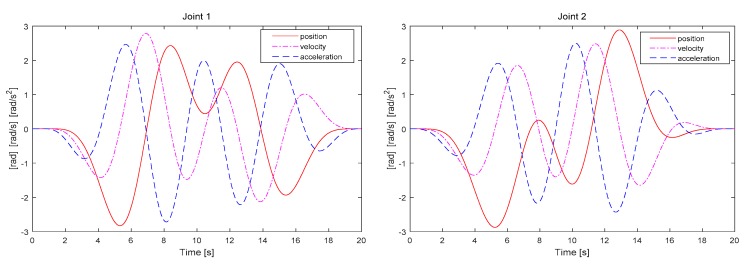
Optimal trajectory found through multi-criteria embedded optimization.

Similar to the optimization process of *F*_1_ and *F*_2_, the nonlinear constraint optimization function “Fmincon” was also used in the MATLAB R2017a environment. This optimization tool adopts the sequential quadratic programming (SQP) algorithm to solve the quadratic programming sub-problem in each step of the iteration process and further update the Lagrangian Hessian matrix until the value of objective function converges to the minimum and the optimization results satisfy the whole constraint space. In order to improve the optimization precision, we extend the iteration times and the error tolerance to enough to search for the solutions within the constraint space, such as “MaxFunEvals” or “TolFun,” which is set as 5000, 1e-10, respectively. Moreover, the *F*_2_ criterion in the constraint condition must satisfy the target value *ω*_2_ = −75, and the optimization must satisfy the Cramér–Rao lower bound in [10]. Figure 5 exhibits the influence of the number of points used for representing the trajectory on *Cond(F)*. The final optimization converges to a certain value. The number of trajectory points is affected by some evaluation termination conditions in the optimization function. The evaluation conditions can be modified to obtain the optimal solution convergence. Figure 6 depicts an optimization trajectory obtained through the proposed method. The joint position, velocity, and acceleration correspond to the independent motion parameters of the actual 2-DOF manipulator.

### 4.4. Experimental Procedure

The parameter identification process of the 2-DOF manipulator was conducted as follows:

Experimental design. Some optimization trajectories were obtained in accordance with the optimization criteria discussed previously. To achieve an improved comparison, the optimal trajectory obtained from the same starting point was selected to satisfy the experiment requirements.

Data acquisition. The controller was equipped with the PID control law to achieve the motion. The trajectory was derived from the abovementioned optimization results (i.e., 11 harmonic functions per joint). During the actual movement, the sampling interval was 0.01 s, and the duration of one period was 20 s. To improve the signal-to-noise ratio, each trajectory continued to move for 320 s, and data of 15 consecutive periods were used for mean filtering. Torque information was collected using torque sensors, position information using a motor incremental encoder, and the velocity and acceleration information through Fourier trajectory differential analysis. All signals were processed through a second-order low-pass digital Butterworth filter to process, and the cutoff frequency was 5 Hz.

Identification. The WLS estimation method was used for the parameter identification of all experimental trajectories. The weights were derived from the noise characteristics of the random trajectory identification results, as described in Section 4.2. The position deviation was close to 0, thereby satisfying the zero-noise hypothesis.

Model validation. The accuracy of the obtained parameter estimates could be verified for a different validation trajectory by comparing the measured torque and the predicted torque based on the model and the measured position data.

## 5. Results

This section provides the results of the experiments described above. In Section 4.2, 12 correlation terms involved in the regression matrix are considered for the experimental design, and the 12 correlation terms are used to identify the dynamic parameters. The identification results and their relative deviations (*σ_i_*) are summarized in Table 4. The relative standard deviation is used to establish a statistical analysis and characterize the quality of parameter estimation as mentioned in [40].

The results presented in Table 4 show that the mean values of the relative standard deviation are lower in each parameter than in other criteria when the *F*_2_ criterion is used because this method finds the estimation with a minimal parameter variance. By contrast, the relative standard deviation mean of each parameter estimated through the multi-criteria embedded optimization method (e.g., *F*_4_) is lower than that based on *F*_1_ and *F*_3_. However, *F*_2_ leads to a higher condition number than other methods, and the identification results are sensitive to noise, as displayed in Table 5. The experiments presented in Table 5 are deliberately designed to increase or decrease the torque residual noise. This process is allowed because obtaining fully determined noise is infeasible in the actual experiment. The results show that *F*_4_ is more robust than *F*_2_. In addition, the relative standard deviation of individual parameters obtained through each method is relatively high because the cited method fails to handle the unmodeled dynamics properly and consider the analytic derivative obtained after the trajectory reparameterization, thus resulting in the difference with the actual parameters. Evidently, all estimated parameters have physically feasible values.

**Figure 7:**
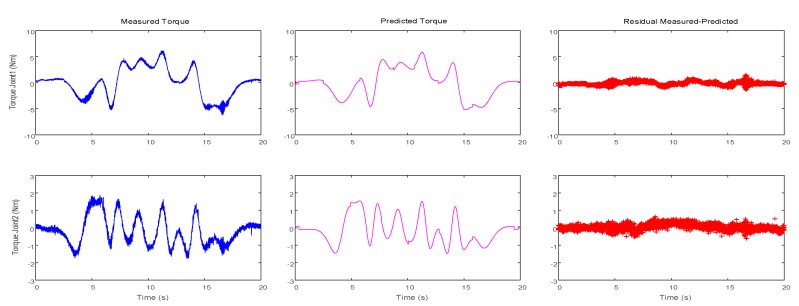
Measured, predicted, and residual torques for the two joints.

**Figure 8:**
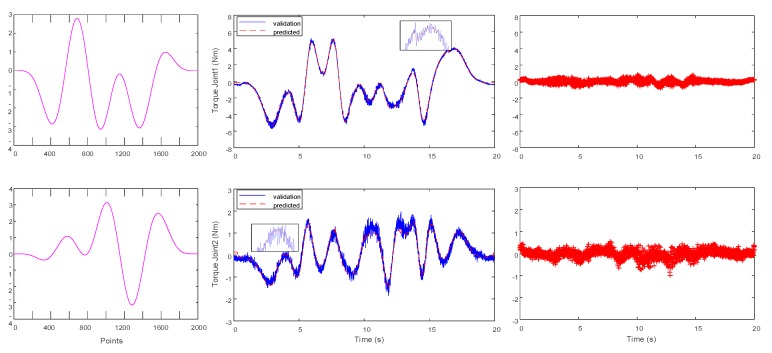
Measured position and torque and predicted and residual torques of the validation experiment.

Parameters obtained through the multi-criteria embedded optimization are used to generate dynamic trajectories, and then the measured torques are compared with the predicted torques, as displayed in Figure 7. Compared with the identification results of the optimized trajectory presented in Figure 4, the residual torque value is smaller than that of the latter, that is, σ_1_ = 0.0516 N^2^m^2^, and σ_2_ = 0.0161 N^2^m^2^. Despite a peak error in the predicted torque curve when the speed or acceleration is reversed, this result is reasonable due to assembly clearance or flexibility. In addition, to verify the accuracy of the identification model, an optimization trajectory that is different from *F*_4_ is selected in Section 4.2 for verification. The verification results are illustrated in Figure 8. Notably, both curves are close. Table 6 presents the root mean square (RMS) of the measured and predicted residuals.

## 6. Discussion and Conclusions

To achieve an ideal model control effect and improve human–robot interaction abilities, an accurate dynamic model must be designed. The experimental design, as an important part of parameter identification, must optimize the excitation trajectories. In this research, some optimization criteria for dynamic parameter identification experiments are evaluated. The results indicated that adopting only *F*_1_ or *F*_2_ criteria will significantly affect the optimization results of the opposition criterion. Although the multi-objective optimization criterion has solved the problem, this criterion was significantly affected by selecting the goal values. Combined with the advantages and disadvantages of each criterion, the multi-criteria embedded optimization method was innovatively proposed. The main contribution is to reduce the DOF of the goal values by adding the criterion to the nonlinear optimization constraints. Furthermore, the optimization results are converted to be mainly restricted by a single time. Considering the motion coupling characteristics of the joint with multiple DOFs, a 2-DOF platform was selected. Based on the optimization simulation and actual experiments, the results showed that the proposed method has achieved the rationalization of the multi-criteria optimization compared with only a single criterion optimization, and the robustness was higher than the multi-objective optimization criterion. The verification experiments presented that the identification results are considerable, and the proposed approach can be considered a suitable procedure for designing the exciting trajectories and improving the results of parameter identification. This method is not only limited to serial robots but also applicable to parallel and exoskeleton robots.

Most existing parameter identification methods focus on off-line identification. However, considering the mechanical wear, aging, temperature, and other nonlinear factors during the use of robots, real-time online identification has become a trend. Therefore, as mentioned in the literature [15,19,41,42,43,44,45], various optimization algorithms and intelligent control theories for improving identification effects must be combined to enable the robots to have the ability of autonomous identification and learning to achieve an efficient and accurate motion control, which have wide applications. In addition, future work will be expanded to include additional DOFs, such as the commonly used 6-DOF or redundant arms, rather than only a 2-DOF manipulator. Furthermore, non-planar motion planning and human–robot interaction control will be realized, and the nonlinear factors, such as flexibility and assembly clearance, will be further investigated.

## Figures and Tables

**Table 1:** Some criteria for the experiment optimization design.

No.	Criteria	References	Frameworks
1	$\frac{1}{\lambda_{\min}(F^{T}F)}$	Armstrong [33]	deterministic
2	Cond(F)	Gautier and Khali [24]	deterministic
3	$\omega_{1}Cond(F)+\omega_{2}\frac{F_{\max}}{F_{\min}}$	Gautier and Khali [24]	deterministic
4	$\omega_{1}Cond(F)+\omega_{2}\frac{1}{\lambda_{\min}(F^{T}F)}$	Presse and Gautier [34]	deterministic
5	$-\log(\det(F^{T}\Sigma^{-1}F))$	Swevers et al. [10]	statistical (*D*-optimality)
6	$\frac{1}{\prod_{g=1}^{N_{b}}F_{g}^{s}}$	Jingfu et al. [25]	deterministic (Hadamard’s inequality)
7	$\left\{ \begin{array}{l} cond(F)-\omega_{1}\gamma \leq F_{1}^{*}\\ -\log(\det(F^{T}\Sigma^{-1}F))-\omega_{2}\gamma \leq F_{2}^{*} \end{array}\right.$	Miguel et al. [37]	statistical

**Table 2:** Values of the objective function for the exciting trajectories.

Trajectories	Optimization Criteria					
	*F*_1_ = Cond(*F*)		*F*_2_ = −log(det(*F^T^*Σ^−1^*F*)^−1^)		*F*_3_ = multi-objective	
	*F*_1_	*F*_2_	*F*_1_	*F*_2_	*F*_1_	*F*_2_
1	8.9564	−46.8065	14.0528	−75.3467	7.9318	−75.6224
2	4.5228	−56.7127	9.9266	−74.1939	7.8595	−76.3168
3	5.4290	−53.1933	9.236	−69.6214	7.9149	−75.7974
4	4.0043	−58.1891	10.8305	−77.0334	7.9156	−75.9591

**Table 3:** Influence of goal value selection on the optimization results.

Trajectories	Goal Weight Values					
	*F*_3_ *ω*_1_ = 5, *ω*_2_ = −70		*F*_4_ *ω*_2_ = −75		*F*_4_ *ω*_2_ = −70	
	*F*_1_	*F*_2_	*F*_1_	*F*_2_	*F*_1_	*F*_2_
1	5.0354	−69.5180	6.9067	−75.0035	6.5072	−70.01
2	4.9852	−70.2078	7.8303	−75.1107	5.0119	−70.0229
3	5.0671	−69.0545	8.2943	−75.0018	5.5365	−70.0113
4	5.0431	−69.396	7.7889	−75.0008	4.8974	−70.0218

**Table 4:** Identified dynamic parameters and their respective deviations of the 2-DOF robot.

Index	*F*_1_		*F*_2_		*F*_3_		*F*_4_	
	*p_b_*	*σ_i_*	*p_b_*	*σ_i_*	*p_b_*	*σ_i_*	*p_b_*	*σ_i_*
1	0.0157	1.99	0.0235	0.94	0.1161	0.56	0.1088	0.81
2	0.0894	0.2	0.0241	0.22	0.0216	0.95	0.0126	1.3
3	0.0023	9.5	0.0017	4.5	0.0363	0.57	0.0329	0.86
4	4.1514	0.009	3.9575	0.011	4.0847	0.036	4.0530	0.049
5	1.2168	0.018	1.2257	0.017	1.2357	0.08	1.2641	0.11
6	0.1253	0.34	0.0918	0.38	0.0146	6.91	0.0742	1.85
7	0.1982	0.087	0.0675	0.46	0.1390	0.91	0.2511	0.69
8	0.1123	0.37	0.0429	0.55	0.0273	3.37	0.0259	4.84
9	0.0692	0.21	0.1072	0.23	0.0767	1.65	0.0930	1.84
mean		1.41		0.81		1.67		1.37

**Table 5:** Comparison of sensitivity with the noise of *F*_2_ and *F*_4._

Noise (σ_1_, σ_2_)		*p_b_*								
*F*_2_	(5.0832,0.0499)	0.0235	0.0241	0.0017	3.9575	1.2257	0.0918	0.0675	0.0429	0.1072
	(0.0516,0.0161)	0.0458	0.0065	0.0189	3.9864	1.2145	0.0340	0.0972	0.0533	0.1111
	(8,1)	0.0384	0.0123	0.0132	3.9776	1.2161	0.0526	0.0876	0.0486	0.1083
*F*_4_	(5.0832,0.0499)	0.1088	0.0126	0.0329	4.0530	1.2641	0.0742	0.2511	0.0259	0.0930
	(0.0516,0.0161)	0.1072	0.0100	0.0313	4.0530	1.2492	0.0747	0.2509	0.0280	0.0923
	(8,1)	0.1079	0.0113	0.0321	4.0528	1.2579	0.0745	0.2510	0.0269	0.0972

**Table 6:** Root mean square (RMS) of the measured and predicted residuals (Nm).

Joints	*F*_4_	Validation
Joint1	0.227	0.235
Joint2	0.127	0.195
